# Light pollution: time to consider testicular effects

**DOI:** 10.3389/ftox.2024.1481385

**Published:** 2024-09-16

**Authors:** Peter Y. Liu

**Affiliations:** Division of Endocrinology, Department of Medicine, David Geffen School of Medicine at UCLA, Harbor-UCLA Medical Center and Genomics Institute, The Lundquist Institute, Torrance, CA, United States

**Keywords:** Leydig cell, testosterone, circadian, fertility, clock

## Abstract

Technological advances have led to a modern-day lighting and smartphone revolution, with artificial light exposure at night increasing to levels never before seen in the evolutionary history of living systems on Earth. Light as a pollutant, however, remains largely unrecognized, and the reproductive effects of light pollution are mostly if not entirely unconsidered. This is despite the reproductive system being intricately linked to metabolism and the circadian system, both of which can be disturbed even by low levels of light. Here, we aim to change this perspective by reviewing the physiological and pathophysiological mechanisms by which light exposure alters the intricate hormonal, metabolic and reproductive networks that are relevant to reproductive toxicology. Nascent human studies have recently identified the photoreceptors responsible for the light dose relationship with melatonin suppression and circadian re-entrainment, directly shown the association between the alignment of light-dark cycles with activity-rest cycles on metabolic health and provided proof-of-principle that properly timed blue light-enriched and blue light-depleted delivery can accelerate circadian re-entrainment. With these advances, there is now a need to consider testicular effects of light pollution.

## Introduction

Artificial light at night (ALAN) was first officially recognized to be an environmental pollutant in 2020, yet awareness of its existence and hazards remains low (The Lancet Regional Health-Europe, 2023). There is a pressing need to promote public awareness of the potential health and reproductive hazards of light pollution as ambient lighting continues to brighten, the default spectral properties of newer lighting remains detrimental, and smartphone and screen-based device use at bedtime is still prevalent and results in unwanted non-ambient light exposure at a critical time at night. Indoor and outdoor ambient lighting has progressively increased since the turn of the century because of the widespread utilization of light emitting diode (LED) technology owing to its longevity, efficiency and reduced cost (Falchi et al., 2016). LEDs typically have a peak in the shorter wavelength (blue spectral) range, compared with the yellowish light of the moon. In addition, these lighting advances have coincided and been incorporated with multiple other technological advances that have led to the worldwide adoption of smartphones. The use of smartphones at bedtime represents an unwanted indoor nighttime exposure to blue spectral light which has detrimental effects on sleep architecture and melatonin circadian rhythmicity (Hohn et al., 2024).

Light pollution has ecological effects on flora and fauna, public health consequences for human wellbeing, and consumes energy and resources (Falchi et al., 2016). Light pollution is a worldwide phenomenon and is highest for individuals residing in North America, Europe and Asia (Falchi et al., 2016). Its pervasiveness is due in part from skyglow which is a diffuse luminance of the night sky when artificial light is scattered in the atmosphere and can be observed in geographical areas far removed from the original light source. Skyglow is highly relevant because low-level light can still disrupt the circadian system (Usmani et al., 2024). Slightly more than 4 in 5 (i.e. 83%) of the world’s population was already exposed to light pollution in 2016 (Falchi et al., 2016). The extent of light pollution has only increased since then as artificial light irradiance has increased by 10% annually over the last decade, and a greater proportion of individuals live near bright light sources due to continuing urbanization of the world’s population (Falchi et al., 2016; The Lancet Regional Health-Europe, 2023; Zielinska-Dabkowska et al., 2023).

## Light pollution alters environmental rhythms that are coordinated to biological rhythms by cellular clocks

Light pollution alters the environmental 24-hour (i.e., diurnal) rhythm of light (i.e., day) and dark (i.e., night) that is dictated by the earth’s diurnal rotation and creates cycles in temperature on land and the sea-surface that can alter sea currents and produce wind in a periodic fashion (Reeves Eyre et al., 2024). These environmental rhythms result in nutrient rhythms, examples of which include photosynthesis and feeding activities of invertebrates and vertebrates (Bus et al., 2023; Weckström and Salovius-Laurén, 2023). Biological processes that are fundamental to survival exhibit these diurnal patterns and include catabolic and anabolic processes, restorative activities such as sleep, and reproductive programs that have a metabolic cost but are necessary for survival of the species.

Living systems developed timing mechanisms (i.e., cellular clocks) to synchronize and optimize energetic processes controlling metabolism to 24-hour nutrient rhythms. These cellular clocks have been extensively characterized and operate through a transcription/translation negative feedback loop that autoregulates periodicity and the rhythmic expression of clock-dependent genes (Sen and Hoffmann, 2020). The feedforward component of the clock loop is the transcriptional activator, Brain and muscle arnt-like protein-1 (BMAL1) and its binding partner Circadian locomotor output cycles kaput (CLOCK). This complex binds to the E-box response element that drives the expression of several genes, including Period (Per1,2,3) and Cryptochrome (Cry1,2). The negative feedback loop consists of PER and CRY which forms a complex that returns to nucleus and interferes with the BMAL1:CLOCK heterodimer thereby autorepressing its own transcription and creating a self-sustaining rhythm with a periodicity that is near 24 h.

## These cellular clocks are unequivocally present and functional in testicular Leydig cells

The testis contains the components of the cellular clock, but clock gene expression does not oscillate in the whole testis and may not oscillate or be functional in sperm cells – although studies may not have properly considered sperm cell type or stage (Bittman, 2016; Chen et al., 2017; Sen and Hoffmann, 2020; He et al., 2023). In contrast, expression of clock genes oscillate endogenously *in vitro* in primary Leydig cell cultures from diurnal (e.g., goat) (Xiao et al., 2021) and nocturnal (e.g., mouse) species (Bittman, 2016; Chen et al., 2017; Medar et al., 2021) and *in vivo* in Leydig cells purified from testes collected at regular intervals from intact rats across an entire 24-hour day (Baburski et al., 2015; Pavlovic et al., 2022). Immunohistochemistry experiments show staining of BMAL1 unequivocally in Leydig cells only, and its staining intensity oscillates in goat and mouse (Alvarez et al., 2008; Chen et al., 2017; Xiao et al., 2021). Since testicular testosterone is produced only by Leydig cells, these data suggest the possibility that 24-hour rhythms in testosterone serve a biological (likely anabolic) purpose, whereas 24-hour rhythms may not be needed for spermatogenesis since this process requires several months to complete (Sen and Hoffmann, 2020).

## Timed light exposure re-entrains the central circadian pacemaker to the environmental rhythm

In mammals, the central circadian pacemaker (CCP) located in the suprachiasmatic nucleus (SCN) of the hypothalamus orchestrates cellular clocks throughout the body through hormonal and neural signals (Bass and Takahashi, 2010; Liu, 2023). These hormones include cortisol, which is also the main catabolic signal, testosterone, which is the major anabolic signal in man, as well as melatonin. In addition to entraining peripheral clocks to the CCP’s rhythm, melatonin serves an important function to re-entrain the CCP. Other hormone signals, such as cortisol, that entrain peripheral clocks to the CCP’s rhythm cannot serve this function because glucocorticoid receptors are not expressed in the SCN and adrenalectomy has no effect on the SCN clock (Oster et al., 2017). Naturally occurring rhythmic phenomena, defined as zeitgebers, are continuously re-entraining the CCP to the environmental day. The most important zeitgeber is light. Light exposure before bedtime delays melatonin onset, light exposure during usual hours of sleep suppresses melatonin, and either of these effects can interfere with the re-entraining properties of light (Gooley et al., 2011).

Humans are a diurnal species, and the retina adapted to high definition color vision through the fovea, a central zone of the retina packed with three types of cones. In contrast, the rodent model is a nocturnal species, and the rodent retina lacks a fovea and consists predominately of rods, and two types of cones. The human eye in particular has two photoreceptive pathways and five photoreceptors (Zielinska-Dabkowska et al., 2023). This first pathway is the primary optic tract for vision at night (scotopic, mediated by rods), daytime (photopic, mediated by blue, red and green cones) and twilight (mesopic, mediated by both rods and cones). The second is a retinohypothalamic tract that signals light and dark to brain regions that primarily regulate circadian, neuroendocrine and neurobehavioral effects of light through a distinct subset of retinal ganglion cells that express melanopsin and are intrinsically photosensitive with an absorption peak in the short-wavelength (blue) portion of the visual spectrum. Subtypes of these intrinsically photosensitive retinal ganglion cells (ipRGCs) are recognized in humans and other primates, with the M1 subtype responsible for photoentrainment of the circadian clock (Mure, 2021). In addition, rod and cone photoreceptors project to the ipRGCs suggesting that separation of function is not complete and both systems contribute to vision, circadian phase resetting and melatonin suppression. Recently, it has been recognized that the phase resetting response is maximally sensitive to light in the first several minutes of light exposure, and that this is driven largely by cone function (particularly blue cone function). On the other hand, melatonin suppression is driven by cone function (with blue being equal to red and green combined) for the first 1–2 h of light exposure, with ipRGCs being dominant over longer duration light exposures and showing a duration dependency (St Hilaire et al., 2022).

## Rhythms in cortisol synchronize cellular clocks in Leydig cells and many other metabolically relevant organs

Diurnal rhythms in both cortisol and testosterone exist, however cortisol’s rhythm is driven largely by the central circadian pacemaker and is circadian in origin, whereas testosterone’s rhythm is in response to the environment and in particular to the timing of sleep (Kelly et al., 2022). In humans, the constant routine protocol experimentally removes external rhythms of light/dark, wake/sleep, and activity/rest by keeping subjects in constant conditions of dark, wakefulness and rest. Other rhythms, for example, in feeding/fasting, can be removed by uniform distribution, such as by hourly delivery and consumption of identical metabolically-neutral snacks. Under such conditions, 24-hour rhythms observed in blood hormones cannot be from external influences, but must be driven endogenously by the central circadian pacemaker and can properly be termed circadian (Mills et al., 1978; Duffy and Dijk, 2002). Three experiments utilizing constant routine have shown that the diurnal rhythm in cortisol is truly circadian and have further characterized its rhythmicity: acrophase (i.e., cortisol rhythm peak) occurs at the habitual sleep-wake transition in the morning and then cortisol levels progressively decreases to an evening nadir (Uchiyama et al., 1998; Wright et al., 2015; Kelly et al., 2022). In addition to being and because it is circadian in origin, this rhythm also serves to be the central *metabolic* synchronizing signal of the CCP for glucose homeostasis and metabolism.

**FIGURE 1:**
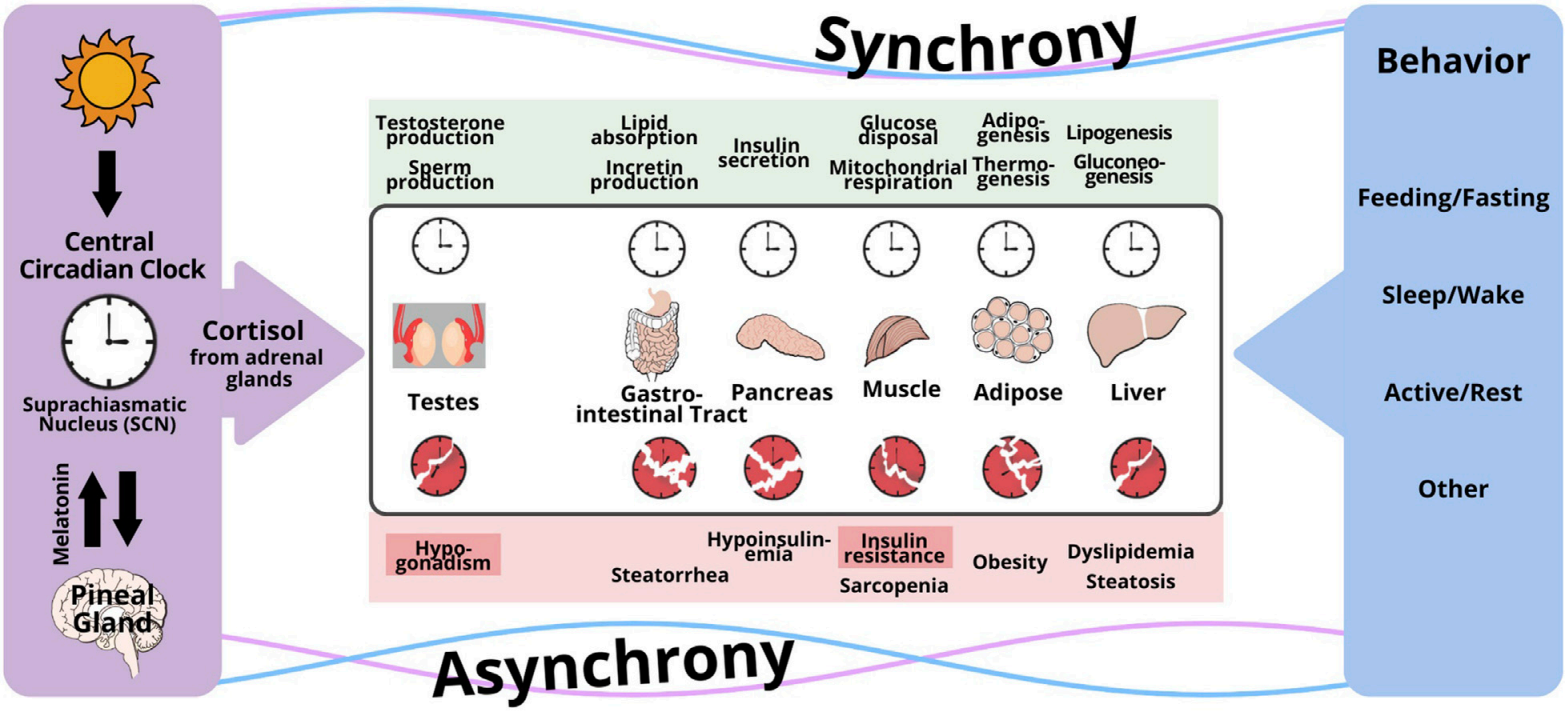
Conceptual framework for the disruption of the circadian regulation of clocks in peripheral organs by conflicting signaling from the central circadian clock and behavioral cycles (feeding/fasting, sleep/wake, activity/rest). The central timing signal is cortisol for Leydig cells in the testes, and peripheral organs responsible for whole body metabolism (gut, pancreas, muscle, fat, liver), whereas peripheral signals governed by behavior include glucagon, insulin, other hormones, metabolites, and others. When peripheral clocks receive conflicting signals, clocks are disrupted and desynchrony occurs, which leads to insulin resistance and metabolic diseases such as obesity and diabetes. Reproduced under a creative commons license with permission from (Liu, 2024b).

Both the glucocorticoid receptor and cellular clock are present in all organs involved in glucose homeostasis: namely, liver, muscle, and adipose tissue which are the principal storage sites for glycogen, protein, and fat, respectively; and the pancreas and gut, which dictate macronutrient absorption (Oster et al., 2017). Timed glucocorticoid administration in rodents and human explant cell systems show that cortisol directly synchronizes peripheral clocks in liver, muscle, and adipose tissue; and putatively also in pancreas and gut (Oster et al., 2017; Wu et al., 2018). The peripheral clocks in these tissues are directly and instantaneously synchronized by cortisol through glucocorticoid response elements present in regulatory regions of core clock genes because removal of glucocorticoid-response elements from the regulatory regions of core clock genes *Bmal1, Cry1, Per1* and *Per2* prevents peripheral clock retiming from glucocorticoid administration (So et al., 2009; Mohawk et al., 2012). Interestingly, cortisol also appears to synchronize cellular clocks in other systems that are metabolically intensive, but necessary for survival such as the Leydig cell which synthesizes testicular testosterone and thereby maintains spermatogenesis. Glucocorticoids directly added to primary Leydig cell cultures induce significant circadian rhythmicity in the transcription of core canonical clock genes *Bmal1*, *Per1* and *Per2* in goat (Xiao et al., 2021) and *Bmal1*, *Per2* and *Cry1* in mouse (Chen et al., 2017). In contrast, melatonin does not alter the transcription of *Bmal1*, *Per1*, *Per2* when added directly to primary rat Leydig cell cultures, and seems to have minor effects *in vivo* (Baburski et al., 2015; Liu, 2024a). When peripheral clocks receive conflicting signals, clocks are disrupted and desynchrony occurs, which leads to insulin resistance and metabolic diseases such as obesity and diabetes: Figure 1 (Bass and Lazar, 2016; Liu, 2019).

## The risks of light pollution – metabolic and testicular

Nighttime light exposure in night shift workers increases rates of obesity, diabetes, cardiovascular disease, hypertension, and other non-metabolic disorders (IARC Working Group on the Identification of Carcinogenic Hazards to Humans, 2020; Zielinska-Dabkowska et al., 2023). Misalignment between light-dark with activity-rest cycles is associated with metabolic harm, as recently demonstrated in a nationally representative US cohort of approximately 7,000 adults from the National Health and Nutrition Examination Survey (Xiao et al., 2023). In this study, activity-rest and light-dark cycles were objectively measured for 7 consecutive days in the field by wrist actigraphy utilizing motion and light sensors. Greater circadian misalignment (reduced strength of coupling between activity and light, and delayed activity-rest relative to light-dark) was associated with diabetes, as well as insulin resistance calculated by the homeostasis model from fasting glucose and insulin in the cross-sectional analysis (Xiao et al., 2023). These relationships persisted after adjusting for important confounders including body mass index, sleep duration and total physical activity. Although this analysis and publication are recent, the data were from the 2011–14 National Health and Nutrition Examination Survey, and indoor ambient light exposure has increased since then because of greater LED adoption, suggesting the possibility that effects may now be even more pronounced. Shift work schedules were not identified in this cohort, so relationships in day, rotating and night shift workers could not be separated. This is relevant because night shift work interferes with the homeostatic regulation of sleep and often leads to sleep loss which can confound findings (James et al., 2017; Pedersen et al., 2022), although this possibility was mitigated by adjustment for individual sleep duration. Furthermore, fewer than 3% of study individuals showed a large phase advance which would be expected with night shift work meaning that the cohort was not engaged in night shift work at the time of assessment. Although persisting effects of past night shift work are still possible, these data nevertheless suggest that smaller misalignments of light-dark with activity-rest that can be observed in any worker (and not the large misalignments observed solely in night shift workers) are associated with important metabolic disorders.

In support of this possibility, an association between morningness chronotype and lower systemic testosterone concentrations has been revealed in small cross-sectional studies (Randler et al., 2012; Jankowski et al., 2019), and some but not all Mendelian randomization studies have shown that chronotype causes low testosterone (Yuan et al., 2023; Lu et al., 2024). These studies that have included mostly, or entirely, men that are not undertaking rotating or night shift work. The distinction is relevant, because night shift work of itself, may not impair male fertility and may not induce hypogonadism in the absence of sleep loss (Liu, 2019; Kelly et al., 2022; Liu, 2024a). Studies of men living close to the Arctic circle have also examined potential reproductive impacts of extremes in daylight duration because known seasonal differences in energy metabolism may contribute to differences in reproductive programs (Malm et al., 2004; Kochan et al., 2008; Arendt, 2012). One study showed significantly higher blood inhibin B concentrations during winter compared with summer in men residing North of the Arctic circle, but no significant difference in semen parameters, FSH and inhibin B when compared during the same season in Norwegian men living north (n = 92) and south (n = 112) of the Arctic circle (Malm et al., 2004). An important limitation of this study is that actual light exposure for each individual was not measured, and artificial light use to mitigate the complete environmental 24-hour darkness of the Arctic winter is likely to have occurred. Furthermore, seasonal daylight duration is accompanied by changes that temperature and other environmental factors that may be confounding.

## Conclusion

Our knowledge of the function of the circadian system in regulating metabolism and the hypothalamic-pituitary testicular axis provides the framework to understand the putative effects of light pollution on reproduction. Direct associations between nighttime light exposure (not simply night shift work) and metabolic function have now been revealed, supported by data showing that chronotype may impact systemic testosterone concentrations, although data are conflicting. Given the strong interrelationships between energy metabolism and fertility (Hansen et al., 2013; Della Torre et al., 2014; Roa and Tena-Sempere, 2014; Service et al., 2023), it is plausible that light pollution could impact fertility – impacts that include adverse effects on puberty from smart phone use at bedtime during adolescence. These issues have received very little attention and now is the time to start considering testicular effects of light pollution because the method to mitigate these effects on circadian alignment are being unveiled. For example, automated LED technology that capitalizes on administering blue-enriched and blue-depleted light in a circadian informed manner can accelerate circadian entrainment to night shift work by 3-fold, and the improve sleep, sleepiness and vigilance (Scott et al., 2024). In the meantime, mitigating the effects of light pollution by light shielding, light minimization appropriate for the task and adaptive lightning should be considered.

## Data Availability

The original contributions presented in the study are included in the article/supplementary material, further inquiries can be directed to the corresponding author.
